# ﻿*Gelidocalamuszixingensis* (Poaceae, Bambusoideae, Arundinarieae), a new species from southern China revealed by morphological and molecular evidence

**DOI:** 10.3897/phytokeys.218.96849

**Published:** 2023-01-10

**Authors:** Cheng-Kun Wang, Rong Guo, Chun-Ce Guo, Guang-Yao Yang, Wen-Gen Zhang

**Affiliations:** 1 Jiangxi Provincial Key Laboratory for Bamboo Germplasm Resources and Utilization, Forestry College, Jiangxi Agricultural University, Nanchang 330045, China Jiangxi Agricultural University Nanchang China; 2 Collaborative Innovation Center of Jiangxi Typical Trees Cultivation and Utilization, Nanchang 330045, China Collaborative Innovation Center of Jiangxi Typical Trees Cultivation and Utilization Nanchang China

**Keywords:** Gramineae, leaf epidermis, phylogeny, SEM, temperate woody bamboo

## Abstract

The genus *Gelidocalamus* T. H. Wen, endemic to southern China, is a small but taxonomically problematic genus of Arundinarieae (Poaceae, Bambusoideae). During field work, a population of *Gelidocalamus* from Zixing, Hunan, was discovered, appearing to be distinct from our previously identified collection. Comparisons of the population of Zixing were performed by using scanning electron microscopy (SEM) and a plastid genome-based phylogeny. Morphologically, it was mostly similar to *G.multifolius*, but differed by culm leaf erect with densely white pubescence, apical branch sheath much longer than the internodes and foliage leaf larger. Phylogenetically, the new species was well-supported as a sister to the clade of *G.multifolius* + *G.tessellatus*, and the above three taxa were clustered in the *Shibataea* clade (IV) of Arundinarieae. Thus, the new species, formally named as *Gelidocalamuszixingensis* W.G.Zhang, G.Y.Yang & C.K.Wang, was described and illustrated herein.

## ﻿Introduction

Arundinarieae (Poaceae: Bambusoideae), i.e., accommodating the temperate woody bamboos, including ca. 581 species in 31 genera (Clark and de Oliveira 2018), is widely accepted as a monophyletic tribe (Guo et al. 2021; Gallaher et al. 2022; Huang et al. 2022), and has been one of the main focuses of botanical research due to its significant ecological and economic value (Triplett 2008). It is mainly distributed in the tropical and subtropical mountains of East Asia, central and southern Africa, Madagascar and eastern North America (Keng and Wang 1996; Soreng et al. 2022). Due to complex allopolyploid history and adaptive radiation events, Arundinarieae has evolved complex and diverse morphological characters, e.g., semelauctant and iterauctant inflorescences, pachymorph and leptomorph rhizomes, and growth habits from solitary to multiple branches, which made it a taxonomically complicated group (Li et al. 2006; Vorontsova et al. 2016; Guo et al. 2021).

As a small but taxonomically problematic genus of Arundinarieae, *Gelidocalamus* T. H. Wen, 1982, containing ca. 11 species (Li et al. 2006; Zhang et al. 2017; Cai et al. 2021), is endemic to southwestern China, and characterized by a set of morphological features including several branches per node, a single foliage leaf on each ultimate branch typically (except *G.multifolius* B. M. Yang, 1986) (Yang 1986), and semelauctant inflorescence. In addition, the new shoots occurring in autumn to winter are also a key feature of *Gelidocalamus* (Keng and Wang 1996; Liu et al. 2017; Nie et al. 2018). Members of *Gelidocalamus* have a relatively narrow distribution in the southern provinces of China and usually occur along ravines under broad-leaved evergreen forest below 1,000 m elevation, except *G.monophyllus* (Yi et B. M. Yang) B. M. Yang, 1989, distributed at 1250 m (Li et al. 2016; Nie et al. 2018). However, some “spring-shoot” species (as opposed to some others that produce shoots in the autumn-winter period), e.g., *G.rutilans* Wen, 1983, *G.subsolidus* W. T. Lin & Z. J. Feng, 1990, *G.solidus* C. D. Chu & C. S. Chao, 1984, and *G.longiinternodus* W. T. Wen & S. C. Chen, 1986, complicate the delimitation of the genus.

Molecular studies of the tribe Arundinarieae have indicated that the conventionally circumscribed *Gelidocalamus* was polyphyletic, and its “spring-shoot” species were nested with members of *Ferrocalamus* Hsueh & Keng f., 1982, *Shibataea* Makino ex Nakai, 1912, *Indocalamus* Nakai, 1925, and other close relatives (Ma et al. 2017; Guo et al. 2019; Qin et al. 2021). Recently, Guo et al. (2021) investigated Arundinarieae using double digest restriction-site associated DNA (ddRAD) sequences, and revealed that six members of *Gelidocalamus* formed a monophyletic clade. These taxa (which may be termed the ‘gelido- taxa’) have identical micromorphological characters (i.e., prominent stomata apparatus surrounded by 8–12 short papillae and a dense waxy covering). On the other hand, other “spring-shoot” members were scattered and grouped with other genera. The “gelido-” members of *Gelidocalamus* are *G.stellatus* T. H. Wen, 1982, *G.tessellatus* T. H. Wen & C. C. Chang, 1982, *G.annulatus* T. H. Wen, 1988, *G.latifolius* Q. H. Dai & T. Chen, 1985, *G.multifolius* and *G.monophyllus*. Two recently reported species *G.xunwuensis* W. G. Zhang & G. Y. Yang, 2017 and *G.fengkaiensis* N. H. Xia & Z. Y. Cai, 2021 appear to also be in this group.

During field work in August 2014, a population of *Gelidocalamus* sp. in Zixing City of Hunan Province, China (25°54'1.75"N, 113°34'9.18"E), was found, and mistakenly identified as *G.multifolius* due to a somewhat similar morphology. In this study, a detailed comparison among the new species, *G.multifolius* and *G.tessellatus*, including characters obtained with scanning electron microscope (SEM) of the foliage leaf epidermis, was made. Moreover, the phylogenetic relationships of the new species including above taxa and allied genera were reassessed based on complete chloroplast genomes.

## ﻿Materials and methods

### ﻿Field investigation and sample collection

Mature bamboo leaves were collected from the individuals of the type localities: *G.* sp from Zixing, Lianping Township of Zixing City in Hunan; *G.stellatus*, Xiazhuang of Jinggang Mountain in Jiangxi; *G.tessellatus*, Maolan of Libo County in Guizhou; *G.multifolius*, Jiuyi Mountain of Ningyuan County in Hunan. Foliage leaves were fixed with the FAA (acetic acid: formalin: ultrapure water: ethanol = 1:2:3:14), and some dried in silica-gel for storage. All voucher specimens were deposited in the herbarium of the College of Forestry, Jiangxi Agricultural University, China (JXAU).

### ﻿Micromorphological observations of foliage leaf epidermis

After cleaning in the ultrasonic cleaner CPX2800H-C (Branson, USA), the middle portion of foliage leaf (5×5 mm) was dried at room temperature, mounted on stubs, and coated with gold sputtering. Using a scanning electron microscope S-4800 (Hitachi, Japan), leaf epidermal characters were observed and photographed. Terminology for epidermal appendages and leaf blades follows previous studies (Ellis 1979; Ellis et al. 2009; Zhang et al. 2014; Leandro et al. 2019).

### ﻿Sequencing, assembly and annotation

Total genomic DNA was isolated from foliage leaves dried over silica-gel by a modified CTAB method (Murray and Thompson 1980). Illumina paired-end (2×150 bp) libraries were constructed and sequenced at Novogene Bioinformatics Technology Co. Ltd. (Beijing, China), and ca. 6 GB raw data for each sample was acquired. To improve assembly accuracy, FastQC 0.11.9 (Andrews 2016) and Fastp 0.12.4 (Chen et al. 2018) were used to filter out unpaired and low-depth reads by using default parameters. Complete chloroplast genomes were assembled using the software GetOrganelle 1.7.4 (Jin et al. 2018) with a range of k-mers of 45, 65, 85, 105 and 121, and the filtered reads were transferred to Bandage (Wick et al. 2015) for chloroplast genome scaffolds connection. Then, chloroplast genome sequences were annotated by using CPGAVAS2 (Shi et al. 2019) and manually checked in Geneious 9.1.4 (Kearse et al. 2012), and illustration of the newly sequenced plastome was drawn in the software Chloroplot with default settings (Zheng et al. 2020).

### ﻿Phylogenetic analysis

To determine the position of the new species, phylogenetic analyses using maximum likelihood (ML) and Bayesian inference (BI) were performed. Besides *G.* sp. from Zixing (OP920758) and *G.multifolius* (OP920759), another 18 complete chloroplast genomes of the tribe Arundinarieae were obtained from the National Center for Biotechnology Information (NCBI, https://www.ncbi.nlm.nih.gov/). *Hsuehochloacalcarea* (C. D. Chu & C. S. Chao) D. Z. Li & Y. X. Zhang, 2018 was selected as outgroup (Genbank accession numbers see the Table 1 for details).

**Table 1.** Information on the 20 complete chloroplast genomes used in this study.

Species	GenBank accession
**Ingroup**	
*Acidosasapurpurea* (Hsueh & T.P.Yi) Keng f.	HQ337793
*Ampelocalamusactinotrichus* (Merr. et Chun) S. L. Chen, T. H. Wen et G. Y. Sheng	MH410123
*Arundinariagigantea* (Walter) Muhl.	NC_020341
*Bergbambostessellata* (Nees) Stapleton	NC_036816
*Gaoligongshaniamegalothyrsa* (Handel-Mazzetti) D. Z. Li	JX513419
*Gelidocalamusmultifolius* B. M. Yang	OP920759
*Gelidocalamustessellatus* Wen & C. C. Chang	NC_024719
*Gelidocalamuszixingensis* W.G.Zhang, G.Y.Yang & C.K.Wang	OP920758
*Himalayacalamusgyirongensis* (Munro) P. C. Keng	NC_043943
*Indocalamussinicus* (Hance) Nakai	NC_036819
*Ravenochloawilsonii* (Rendle) D. Z. Li & Y. X. Zhang	JX513421
*Kurunadebilis* (Thwaites) Attigala, Kaththr. & L.G.Clark	NC_036822
*Oldeaniaalpina* (K.Schum.) Stapleton	NC_036813
*Phyllostachysedulis* (Carriere) J. Houzeau	MW007170
*Pleioblastusamarus* (Keng) Keng f.	MH988736
*Sinoasalongiligulata* (McClure) N.H.Xia, Q.M.Qin & J.B.Ni	NC_036825
*Shibataeachiangshanensis* Wen	NC_036826
*Shibataeakumasasa* (Zollinger ex Steudel) Makino ex Nakai	KU523578
*Thamnocalamusspathiflorus* (Trinius) Munro	JX513425
**Outgroup**	
*Hsuehochloacalcarea* (C.D.Chu & C.S.Chao) D.Z.Li & Y.X.Zhang	KJ496369

After alignment with MAFFT 7.450 (Katoh and Standley 2013), Maximum likelihood (ML) analysis was generated by IQ-TREE (Nguyen et al. 2015), bootstrap analyses were performed with 1,000 replications, and the best-fit BIC model GTR + F + I + G4 was defined by ModelFinder (Kalyaanamoorthy et al. 2017). Bayesian inference (BI) was conducted using MrBayes 3.2.6 (Ronquist et al. 2012) with the same model. 20,000,000 generations were run to ensure average standard deviation of split frequencies (ASDFs) < 0.01 with sampling frequency set as 2,000 generations. Discarding the first 25% burn-in samples, the optimized topology was printed.

## ﻿Results

### ﻿Morphological comparison

**Figure 1.**
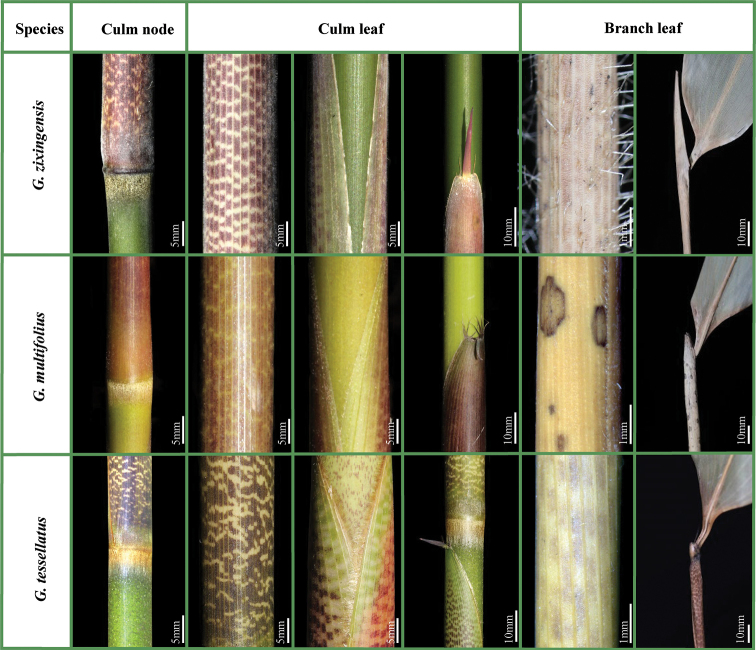
Comparison of key characters among *G.zixingensis*, *G.multifolius* and *G.tessellatus*. The corresponding scales are at the bottom right of the figures.

Compared to *G.tessellatus*, *G.zixingensis* was mostly similar to *G.multifolius* in the habit, the morphological characters and new shoots sprouting season, but can be distinguished by the following morphological characters: (a) a ring of white-gray (vs. yellow-brown) appressed pubescence below each culm node; (b) culm leaf sheaths densely white pubescent (vs. glabrous), with brown patches (vs. pale brown), and erect (vs. reflexed) culm sheath blades; (d) branch leaf sheaths setose (vs. glabrous) and much longer, (>3cm) (vs. slightly longer, < 1cm or as long) as the internodes; foliage leaf blades mesophyll (vs. notophyll). (see Table 2, Fig. 1 for details).

**Table 2.** Morphological comparison among *G.zixingensis*, *G.multifolius* and *G.tessellatus*.

Characters	*G.zixingensis*	*G.multifolius*	*G.tessellatus*
Culm	glabrous, each node with a ring of white-gray appressed trichomes below.	glabrous, each node with a ring of yellow-brown appressed trichomes below.	sparsely setae, each node with a ring of golden appressed trichomes below.
Culm leaf	sheath pubescent with brown patches, sparsely setae near the base, margin sparsely ciliate; oral setae 2–4 pairs; blade erect.	sheath glabrous with pale brown patches, margin sparsely ciliate; oral setae 3–6 pairs; blade reflexed.	sheath glabrous with purple patches, black setae, margin densely ciliate; oral setae 2–3 pairs; blade erect or reflexed.
Branch	sheath papery, densely setae, without black spots, apical branch sheath longer ca. 3 cm than that of the internode.	sheath leathery, with black spots, pubescent, apical branch sheath longer 0.5–1cm than that of the internode.	sheath leathery, without black spots, sparsely setae, apical branch sheath equally or shorter than that of the internode.
Foliage leaf	oral setae 1–3 pairs; blade mesophyll, 23–32×3.2–4.9 cm, lateral veins 6–8 pairs	oral setae weak or absent; blade notophyll, 8–14×1.5–2.5 cm, lateral veins 4–6 pairs	oral setae weak or absent; blade mesophyll, 17–35×3.7–5.4 cm, lateral veins 5–6 pairs

### ﻿Micromorphological comparison of abaxial foliage leaf epidermis

**Figure 2.**
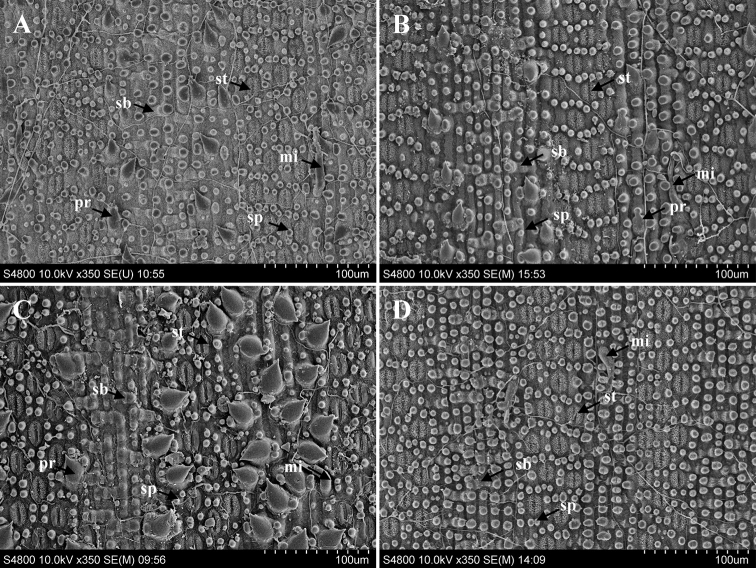
SEM images of the abaxial foliage leaf epidermis **A***G.zixingensis* (Zixing, Hunan, China) **B***G.multifolius* (Ningyuan, Hunan, China) **C***G.tessellatus* (Libo, Guizhou, China) **D***G.stellatus* (Jinggang Mountain, Jiangxi, China). Abbreviations: mi, microhairs; pr, prickles; sb, silica bodies; sp, short papillae; st, stomatal apparatuses.

Epidermal traits of the foliage leaf, e.g., short papillae, microhairs, silica bodies and prickles, can be clearly identified under the scanning electron microscope (Fig. 2). Main characters shared by the four selected taxa were: (a) exposed stomatal apparatus, densely covered with white wax and surrounded by 8–10 short papillae; (b) bicellular microhairs, of which the apical one was withered; (c) saddle-shaped silica body, mainly distributed between veins (Table 3). Prickles were sparsely distributed between the veins in *G.zixingensis* and *G.multifolius*, and more densely distributed in the *G.tessellatus*, while no prickles were observed in *G.stellatus*. Besides, the number of stomatal rows was different, e.g., 3 in *G.tessellatus*, 3 or 4 in *G.stellatus*, 4 in *G.multifolius*, but 5 in *G.zixingensis*.

**Table 3.** Micromorphological comparison among four taxa in the study.

Characters	*G.zixingensis*	*G.multifolius*	*G.tessellatus*	*G.stellatus*
Stomatal apparatus	5 rows distributed between the veins	4 rows distributed between the veins	3 rows distributed between the veins;	3 or 4 rows distributed between the veins
Papillae	8–10 surrounded the stomatal apparatus			
Microhair	bicellular, apical cell withered			
Prickle	sparsely distributed on the veins	sparsely distributed on the veins	relatively densely distributed on the veins	absent
Silica body	saddle-shaped			

### ﻿Phylogenetic analyses based on complete chloroplast genomes

**Figure 3.**
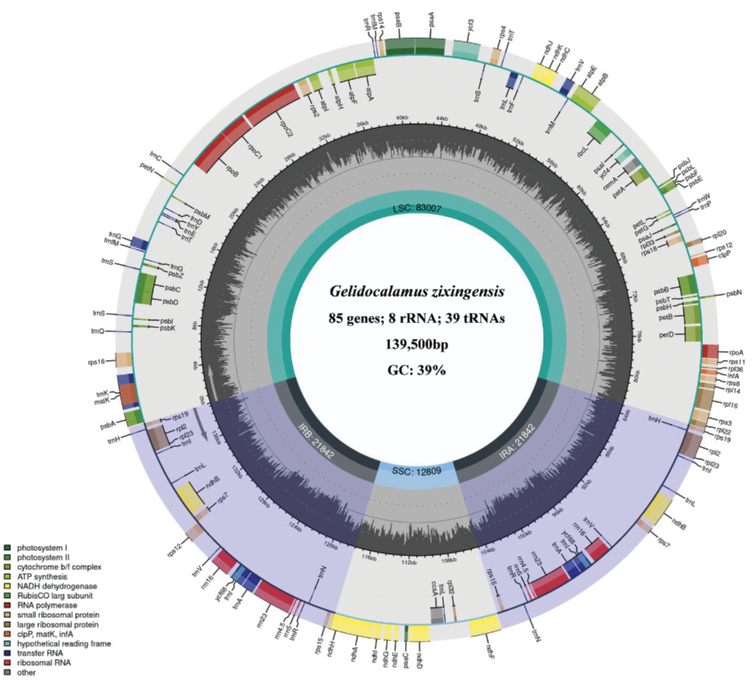
Complete chloroplast genome map of the *Gelidocalamuszixingensis*.

The complete chloroplast genome of *Gelidocalamuszixingensis* was 139,500 bp in length, comprising a large single copy (LSC) region of 83,007 bp, a small single copy (SSC) region of 12,809 bp and two inverted repeat (IR) regions of 21,842 bp, and its GC content was 39%. The chloroplast genome contained 132 genes, including 85 protein-coding genes, 39 transfer RNAs and 8 ribosomal RNAs (Fig. 3), and the total length of the aligned plastid matrix data was 143,738 bp.

Compared to that in *G.zixingensis*, the total length of chloroplast genome of *G.multifolius* and *G.tessellatus* was longer (>200bp), and the differences were mainly in the LSC region (Table 4). Moreover, in comparing the chloroplast genomic variant loci of the three species, a total of 123 SNPs (Single-Nucleotide Polymorphism) and 44 INDELs (Insertion-Deletion) were identified, of which 92 SNPs (74.7%) and 40 INDELs (90.9%) were located in the LSC region.

**Table 4.** Comparison of complete chloroplast genomes of three taxa in the study.

Characters	*G.zixingensis*	*G.multifolius*	*G.tessellatus*
Total length	139,500	139,745	139,712
LSC region	83,007	83,252	83,220
SSC region	12,809	12,809	12,808
IR region	21,842	21,842	21,842
Total genes	132	132	132
CDS	85	85	85
tRNA	39	39	39
rRNA	8	8	8

**Figure 4.**
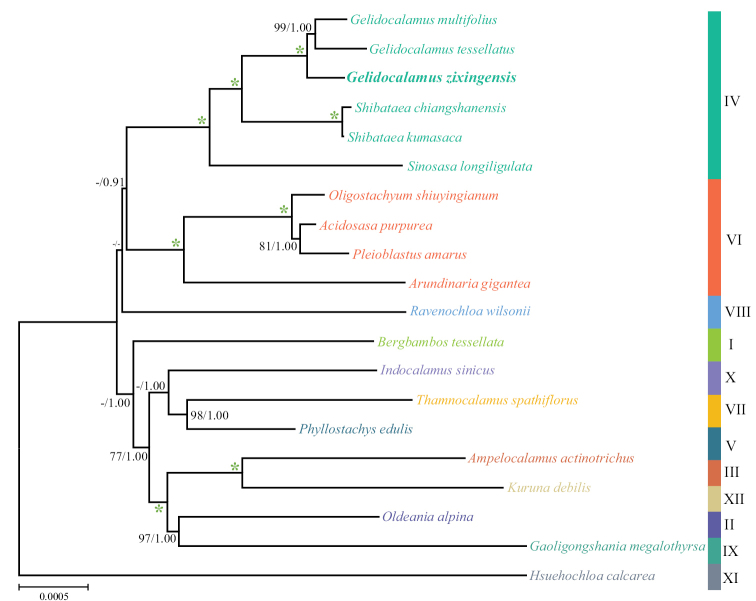
Phylogenetic consensus tree of the *Gelidocalamuszixingensis* based on plastid genome dataset with maximum likelihood and Bayesian analyses. Only bootstrap values (BS) ≥ 75% and posterior probabilities (PP)≥0.75 are indicated at each node, otherwise dashes. The green asterisk indicates support of 100% BS and 1.00 PP. The letters represent the major chloroplast marker-based clade (I-XII) in which the selected taxa are located.

The majority-rule consensus tree with both maximum likelihood (ML) and Bayesian inference (BI) analyses was shown in Fig. 4. Arundinarieae is well-supported as a monophyletic entity, finely divided into 12 lineages (I–XII). There is high support for the *G.zixingensis* being a sister to the *G.multifolius* + *G.tessellatus* clade (bootstrap value of 100% in ML analysis and posterior probability of 1.0 in BI analysis), and the above 3 species were clustered with members of *Shibataea* e.g., *S.chiangshanensis* and *S.kumasaca*, member of *Sinosasa*, e.g., *S.longiligulata*, to form the IV clade (bootstrap value of 100% in ML analysis and posterior probability of 1.0 in BI analysis).

### ﻿Taxonomic treatment

#### ﻿ Gelidocalamus zixingensis

Taxon classificationPlantaePoalesPoaceae

W.G.Zhang, G.Y.Yang & C.K.Wang sp. nov.

51FE44B8-4B5B-5DCE-8D99-DEED3EC11BA6

urn:lsid:ipni.org:names:77311677-1

Figs 5 , 6

##### Diagnosis.

The new species is morphologically similar to *G.multifolius*, but differs by having densely white pubescence (vs. glabrous) on the culm leaf sheaths, culm leaf blades erect (vs. reflexed); apical branch sheaths much longer (vs. slightly longer or equilong) than the internodes; foliage leaf blades mesophyll (vs. notophyll).

##### Type.

China. Hunan, Zixing County, Lianping Township, Chengkang Village, under the forest, 25°54'1.75"N, 113°34'9.18"E, elev. ca. 594 m, 18 Oct. 2015, *W.G. Zhang* et al. LPC031 (holotype: JXAU!)

##### Description.

Rhizomes leptomorph. Culms 1.7–4.2 m, 3.5–10 mm in diameter; erect, apically slightly nodding; internodes initially covered with white pubescence, ca. 14–35 cm long, wall 0.6–1.9 mm thick; each node with a ring of white-gray appressed pubescence below sheath scar; branching intravaginal, arising from 5^th^ node above ground, ca. 4–11 (16) branches per node; branches equal or subequal, ca. 5–30 cm long. Culm leaves sheaths persistent, 12–19 cm, culm leaf sheath abaxially with brown patches, densely white pubescent and sparsely setose near the base; culm leaf blade erect, linear-lanceolate, 0.5–2 cm long, 2 mm wide, apex acuminate, base blunt or truncate, ca.1/3 as wide as sheath apex, oral setae 2–4 on each side of the sheath apex, ca. 4 mm long; auricles absent; ligule truncate, ca. 0.5 mm high, scabrous. Branch sheath papery, white setose, without black spots, margins ciliate; sub-apical branch sheath ca. 3 cm beyond the internode. Foliage leaves usually solitary on ultimate branches; ligule truncate, ca. 1 mm, scabrous; auricles absent; oral setae 1–3 pairs straight or curved; leaf blade broadly lanceolate, usually 23.4–32.5×3.2–4.9 cm, lateral veins 6–8 pairs, abaxial surface basally pubescent, base cuneate and asymmetrical, margins serrulate and slightly revolute near base.

##### Phenology.

New shoots in October.

##### Etymology.

The species epithet refers to the locality of the type specimen: Zixing County, Hunan, China.

##### Vernacular names.

Zī Xīng Duăn Zhī Zhú (Chinese pronunciation), 资兴短枝竹 (Chinese name).

##### Distribution and habitat.

To date, this species has only been found under evergreen broad-leaved forest along river banks at 500–600 m in Chengkang Village, Lianping Township, Zixing County. Species growing in the surrounding area include *Quercusmyrsinifolia* Blume, 1871 (Fagaceae), *Araliachinensis* L., 1868 (Araliaceae), *Euryajaponica* Thunb., 1783 (Pentaphylacaceae), and *Liriopespicata* (Thunb.) Lour., 1790 (Asparagaceae).

**Figure 5.**
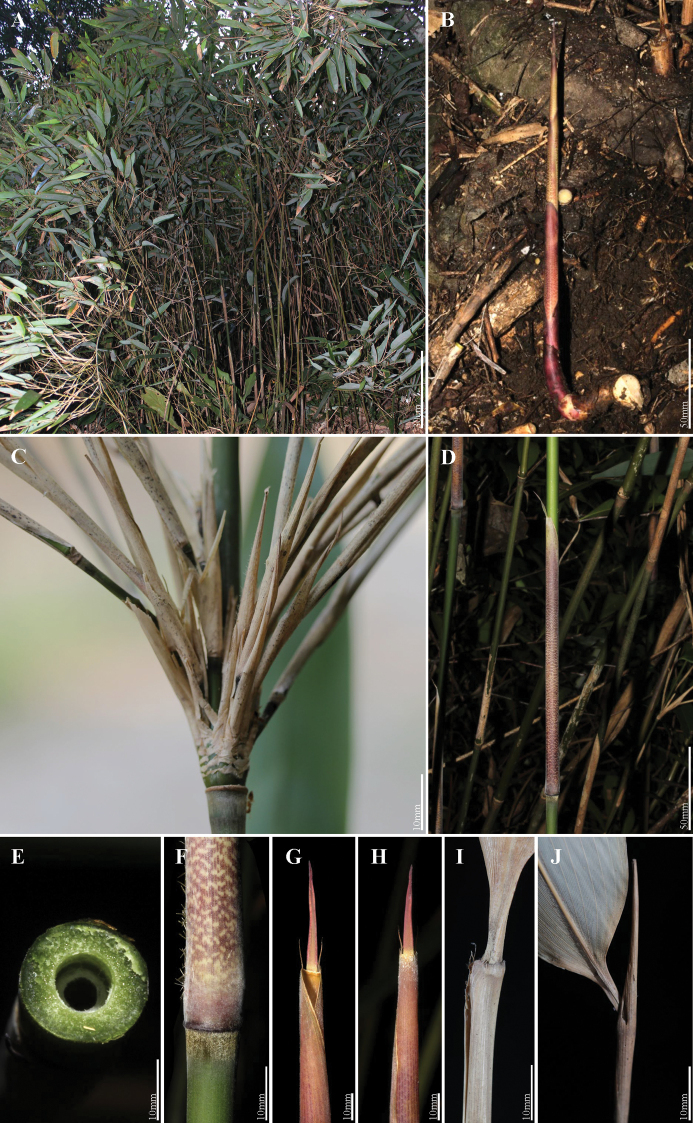
*Gelidocalamuszixingensis***A** habitat **B** new shoot **C, J** branch and its leaf sheath **D, F, G, H** culm and its leaf sheath **E** transection of culm and pith-cavity **I** leaf Sheath.

**Figure 6.**
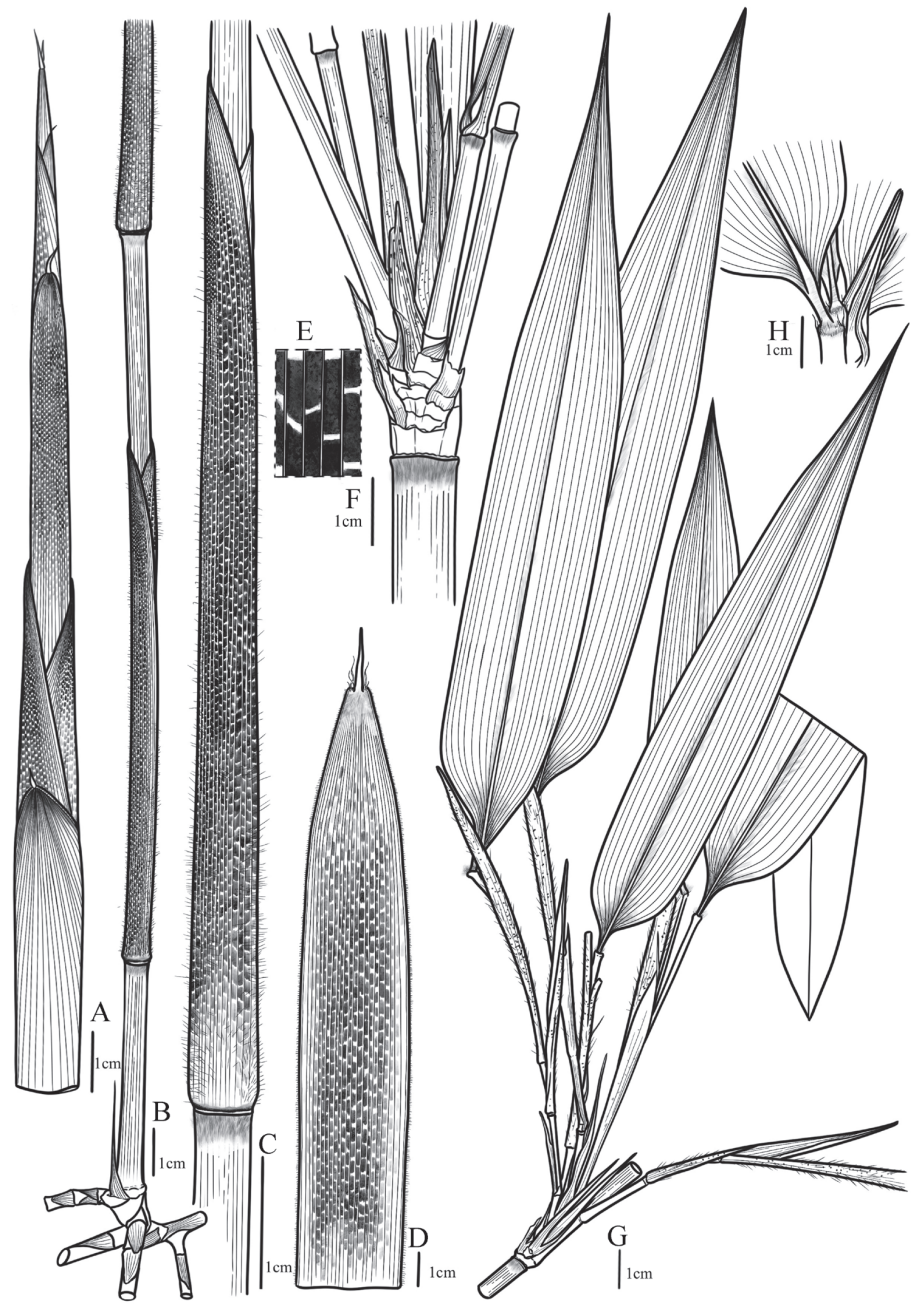
*Gelidocalamuszixingensis***A** new shoot **B** leptomorph rhizomes and culm **C–E** culm and its sheath, brown patches **F–H** branch and its sheaths, leaf and leaf sheath.

### ﻿Morphological key of the species of *Gelidocaldums* of China

**Table:** 

1	Culm internodes glabrous	**2**
–	Culm internodes hairy	**5**
2	Culm leaf sheaths glabrous	**3**
–	Culm leaf sheaths pubescent with sparse setae	***Gelidocalamuszixingensis***
3	Culm leaf sheath margins glabrous or one margin ciliate; oral setae absent or small	**4**
–	Culm leaf sheath margins ciliate; oral setae 3–6 pairs	***Gelidocalamusmultifolius***
4	Oral setae of culm leaves 1–2 pairs, small; branch sheath margins glabrous; branch sheaths with black spots	***Gelidocalamusstellatus***
–	Oral setae of culm leaves none or several; branch sheath margins ciliate at one side, the other side glabrous or apically ciliate; branch sheaths without black spots	***Gelidocalamusfengkaiensis***
5	Culm leaf sheaths covered with appressed brown short hairs	**6**
–	Culm leaf sheaths covered with white erect thin hairs	**8**
6	Culms up to 5m tall, more than 1cm in diam	***Gelidocalamustessellatus***
–	Culms less than 4m, less than 1cm in diam	**7**
7	Culm leaf sheaths with white villus, margins with cilia	***Gelidocalamusmonophyllus***
–	Culm leaf sheaths hairless, margins glabrous	***Gelidocalamusxunwuensis***
8	Culm leaf sheath margins densely ciliate, oral setae 1 pair, foliage leaves 1(–2) per ultimate branch, lateral veins 6–9 pairs	***Gelidocalamuslatifolius***
–	Culm leaf sheath margins hairless, oral setae 2–3 pairs, foliage leaves 1–3 per ultimate branch, lateral veins 4–6 pairs	***Gelidocalamusannulatus***

## ﻿Discussion

Morphologically, although its inflorescence is not seen so far, *G.zixingensis* is undoubtedly a member of the genus *Gelidocalamus*, because it possesses all key characters of the genus, i.e., leptomorph rhizomes, several branches per node, typically a single foliage leaf on each ultimate branch, semelauctant inflorescence (Wen 1982; Li et al. 2006; Yi et al. 2008). However, it is obviously different from other species of the genus, e.g., conspicuously longer than the internodes and culm leaf densely white pubescent. At first glance, *G.zixingensis* is similar to *G.multifolius* in appearance, but can be distinguished by a ring of white-gray appressed pubescence below each node, culm sheaths densely pubescent with brown patches, sub-apical branch sheath much longer than the internode, and a single foliage leaf on each ultimate branch.

Previous studies indicated that leaf epidermal features were of taxonomic significance in Bambusoideae (Soderstrom and Ellis 1988; Yang et al. 2008; Zhang et al. 2014; Leandro et al. 2019). According to papilla form and distribution patterns around the stomatal apparatus of the abaxial leaf epidermis, *Gelidocalamus* can be classified into at least three types: (a) short papillae, none on the stomatal apparatus, e.g., *G.stellatus*, *G.multifolius*, *G.tessellatus*; (b) elongate or short papillae overarching the stomata, e.g., *G.subsolidus*, *G.solidus*; (c) many short papillae, completely covering stomatal apparatus, e.g., *G.monophyllus* (Wu et al. 2014). Compared to those of the “spring-shoot” taxa, leaf epidermal characters in the “gelido-” members of *Gelidocalamus* are relatively stable, and can be used as a diagnostic feature. In the present study, epidermal traits of foliage leaf in *G.zixingensis* were found to be consistent with these of six “gelido-” members in *Gelidocalamus* (except *G.monophyllus*, Liu et al. 2017; Nie et al. 2018), and the difference mainly lay in the fact that *G.zixingensis* had 5 rows of stomatal apparatus.

The tribe Arundinarieae was known for its complex phylogenetic relationships. Despite many previous attempts based on different datasets having been made, intractable problems, such as low resolution or heavily conflicting topologies, still arose (Zhang et al. 2012; Yang et al. 2013; Zeng et al. 2014; Ma et al. 2017). Recently, with the wider sampling of Arundinarieae, Guo et al. (2021) provided a robust phylogenetic tree of the tribe, referred from the ddRAD dataset, which was mostly consistent with the morphological data. In the phylogenetic analysis, only six “gelido-” members formed a monophyletic lineage, although all members of *Gelidocalamus* belonged to the leptomorph lineage. Together with our present molecular phylogenetic analysis, we confirm that *G.zixingensis* belongs to the genus *Gelidocalamus*, and it is closely related to *G.multifolius*.

## Supplementary Material

XML Treatment for Gelidocalamus zixingensis

## Supplementary materials

Supplementary material 1 *Gelidocalamuszixingensis* complete chloroplast genome sequences Data type Phylogenetic. https://binary.pensoft.net/file/793695This dataset is made available under the Open Database License (http://opendatacommons.org/licenses/odbl/1.0/). The Open Database License (ODbL) is a license agreement intended to allow users to freely share, modify, and use this Dataset while maintaining this same freedom for others, provided that the original source and author(s) are credited.Cheng-Kun Wang, Rong Guo, Chun-Ce Guo, Guang-Yao Yang, Wen-Gen Zhang
